# Identification of a Monoclonal Antibody That Attenuates Antiphospholipid Syndrome-Related Pregnancy Complications and Thrombosis

**DOI:** 10.1371/journal.pone.0158757

**Published:** 2016-07-27

**Authors:** Chieko Mineo, Lane Lanier, Eunjeong Jung, Samarpita Sengupta, Victoria Ulrich, Anastasia Sacharidou, Cristina Tarango, Olutoye Osunbunmi, Yu-Min Shen, Jane E. Salmon, Rolf A. Brekken, Xianming Huang, Philip E. Thorpe, Philip W. Shaul

**Affiliations:** 1 Center for Pulmonary and Vascular Biology, Department of Pediatrics, University of Texas Southwestern Medical Center, Dallas, Texas, United States of America; 2 Department of Internal Medicine, University of Texas Southwestern Medical Center, Dallas, Texas, United States of America; 3 Department of Medicine, Hospital for Special Surgery, Weill Cornell Medical College, New York, New York, United States of America; 4 Department of Pharmacology and the Hamon Center for Therapeutic Oncology Research, University of Texas Southwestern Medical Center, Dallas, Texas, United States of America; 5 Department of Surgery, University of Texas Southwestern Medical Center, Dallas, Texas, United States of America; Medical Faculty, Otto-von-Guericke University Magdeburg, Medical Faculty, GERMANY

## Abstract

In the antiphospholipid syndrome (APS), patients produce antiphospholipid antibodies (aPL) that promote thrombosis and adverse pregnancy outcomes. Current therapy with anticoagulation is only partially effective and associated with multiple complications. We previously discovered that aPL recognition of cell surface β2-glycoprotein I (β2-GPI) initiates apolipoprotein E receptor 2 (apoER2)-dependent signaling in endothelial cells and in placental trophoblasts that ultimately promotes thrombosis and fetal loss, respectively. Here we sought to identify a monoclonal antibody (mAb) to β2-GPI that negates aPL-induced processes in cell culture and APS disease endpoints in mice. In a screen measuring endothelial NO synthase (eNOS) activity in cultured endothelial cells, we found that whereas aPL inhibit eNOS, the mAb 1N11 does not, and instead 1N11 prevents aPL action. Coimmunoprecipitation studies revealed that 1N11 decreases pathogenic antibody binding to β2-GPI, and it blocks aPL-induced complex formation between β2-GPI and apoER2. 1N11 also prevents aPL antagonism of endothelial cell migration, and in mice it reverses the impairment in reendothelialization caused by aPL, which underlies the non-thrombotic vascular occlusion provoked by disease-causing antibodies. In addition, aPL inhibition of trophoblast proliferation and migration is negated by 1N11, and the more than 6-fold increase in fetal resorption caused by aPL in pregnant mice is prevented by 1N11. Furthermore, the promotion of thrombosis by aPL is negated by 1N11. Thus, 1N11 has been identified as an mAb that attenuates APS-related pregnancy complications and thrombosis in mice. 1N11 may provide an efficacious, mechanism-based therapy to combat the often devastating conditions suffered by APS patients.

## Introduction

The antiphospholipid syndrome (APS) is an autoimmune disease characterized by the production of antiphospholipid antibodies (aPL) that promote vascular thrombosis and also complications during pregnancy including fetal loss [1–4]. In addition to its occurrence in individuals without an underlying disorder, APS is prevalent in patients with systemic lupus erythematosus (SLE), with as many as 34% of lupus patients having circulating aPL [2,3,5]. Along with arterial and venous thrombosis and pregnancy complications, patients with APS have increased incidence of non-thrombotic vascular occlusion and greater risk of coronary artery disease, myocardial infarction, and stroke [6–9]. Accumulating evidence to date indicates that interactions between circulating aPL and cell surface molecules of target cells such as endothelial cells and trophoblasts underlie the clinical disorders in APS [1,4]. Although recent studies have begun to elucidate the molecular basis for APS, currently available therapies are limited to the prevention of thrombosis through long-term anticoagulation, which is inconvenient, expensive, and fraught with complications including hemorrhage and osteoporosis [10–12]. More effective treatment strategies are needed that are based upon the molecular mechanisms by which aPL cause cellular dysfunction.

Recent studies in humans and in animal models have revealed that most pathologically relevant aPL are directed against certain epitopes in the phospholipid-binding protein β2-glycoprotein I (β2-GPI) [1,13]. β2-GPI is a plasma protein comprised of five distinct domains designated I-V, and it binds to cell surface anionic phospholipids through Domain V [14,15]. Elevated levels of certain circulating anti-β2GPI antibodies are positively associated with both thrombosis and reproductive failure in APS patients [16]. In particular, antibodies to an epitope within Domain I of β2-GPI are highly associated with disease severity [16–19]. Paralleling the human condition, the introduction of particular circulating anti-β2-GPI antibodies in mice, either by passive or active immunization, induces thrombus formation and fetal loss [20–22]. Moreover, β2-GPI^-/-^ mice are protected from pregnancy loss induced by aPL [23]. We previously discovered in studies in culture and in mice that aPL binding to β2-GPI initiates apolipoprotein E receptor 2 (apoER2)-dependent signaling in endothelial cells and in placental trophoblasts that ultimately promotes thrombosis and fetal loss, respectively [24,25]. Blockade of β2-GPI recognition by aPL may provide a mechanism-based means to intervene therapeutically in APS patients.

In the current work, we sought to identify a monoclonal antibody (mAb) to β2-GPI that provides protection from the adverse actions of aPL. In a screen measuring endothelial NO synthase (eNOS) enzyme activity in intact cultured endothelial cells, we found that whereas aPL potently antagonize eNOS, the anti-β2-GPI mAb 1N11 does not mimic aPL inhibition of eNOS, and instead 1N11 prevents the effect of aPL on the enzyme. We determined the mechanism of action of 1N11, and assessed whether 1N11 preserves normal endothelial and also trophoblast cellular functions in the presence of aPL. In mouse models of APS we then determined how 1N11 impacts the attenuation of endothelial repair by aPL, which underlies aPL-related non-thrombotic vascular occlusion, the promotion of fetal loss by aPL administered during pregnancy, and aPL-induced thrombosis remote from pregnancy.

## Materials and Methods

### Antibody Isolation and Purification

Polyclonal normal human IgG (NHIgG) was obtained from healthy non-autoimmune individuals. Polyclonal aPL were isolated from patients with APS having high-titer aPL Ab (>80 GPL U), thromboses, and/or pregnancy complications [24,26]. The relevant laboratory and clinical features of the patients who provided aPL are provided in the S1 Table. Individuals gave written informed consent before participating in these studies, and all protocols were approved by the Institutional Review Boards of the Hospital for Special Surgery and the University of Texas Southwestern Medical Center. NHIgG and aPL were purified as previously reported [24,26–28]. The anti-β2-GPI mAb 3F8, 2aG4, 3J05 and 1N11 were generated as previously described [24,29–31]. A hybridoma secreting C44, a colchicine-specific mouse IgG2a mAb, was obtained from the American Type Culture Collection (Manassas, VA) and used as a control for murine chimeric 1N11. All antibodies produced in culture supernatants were purified as described previously [29,32].

### Nitric Oxide Synthase (NOS) Activity Assay

Primary bovine aortic endothelial cells (BAEC) were isolated using previously described methods, cultured in EGM2 media (Lonza) and used within 3–7 passages [24,26]. Human aortic endothelial cells (HAEC) (Lonza) were maintained in EGM-2 and used at 3–5 passages. eNOS activation was assessed in intact endothelial cells by measuring the conversion of [^14^C]-L-arginine to [^14^C]-L-citrulline [24]. Briefly, BAEC or HAEC were preincubated with NHIgG or aPL (100 μg/ml), in the presence versus absence of mAb (1N11, 3J05, 3F8, 2aG4 or C44) at the indicated concentrations for 60 min, and eNOS activity was then determined over 15 minutes in the continued presence of NHIgG or aPL and/or mAb, in the absence (basal) or presence of vascular endothelial growth factor (VEGF, 2.4 pmol/l, 100 ng/ml).

### Binding Assay

Monoclonal IgG were biotinylated using an EZ-Link NHS-PEO4-Biotinlation Kit (Thermo Scientific, IL) according to the manufacturer's instructions, and the binding assay was performed as previously reported [29]. Briefly, 96-well plates were coated with phosphatidylserine solution (50 μg/ml dissolved in hexane) for 2h at RT until solvent was evaporated. PBS containing purified human β2-GPI (10 μg/ml, Haematologic Technologies, Inc) was added for 2h and then removed, and biotinylated mAbs at the concentrations indicated in the figure legends were added for 2h at RT. After washing, bound biotinylated IgG was detected by horse radish peroxidase-conjugated streptavidin (1:1000 dilution, Thermo Scientific) followed by the addition of TMB substrate (Thermo Scientific), and binding was quantified by spectrophotometric measurement of absorbance at 450 nm wavelength.

### Crosslinking, Immunoprecipitation, and Immunoblotting

HAEC were treated with subclass-matched IgG control (C44), 3F8 or 1N11 for 1h in M199 medium containing 1% FBS, and the crosslinker DTSSP (1.5mM, Life Technologies) was added for 30min at RT. The reaction was terminated by the addition of stop solution containing 15mM Tris, pH 7.5, and protease inhibitors. After washing, cell lysates were prepared, and immunoprecipitation was performed using anti-ApoER2 polyclonal antibody (1.5 μg/ml, Abcam) and protein A/G agarose beads (Santa Cruz) incubated for 2h at 4°C. After washing, samples were dissolved in SDS PAGE sample buffer containing 62.5 mM Tris-HCL, pH 6.8, 2.5% SDS, and 5% β-mercaptoethanol, and proteins were separated by SDS PAGE. Immunoprecipitated ApoER2 was detected by immunoblot using anti-ApoER2 antibody (Abcam), and co-immunoprecipitated β2GPI was detected using mAb 2aG4.

### Endothelial Cell Migration

BAEC or HAEC were grown to near-confluence in 6-well plates and placed in 1% lipoprotein-deficient serum (LPDS) in Dulbecco’s modified Eagle’s medium (DMEM) (Sigma-Aldrich) for 16 h, and a defined region of cells was removed with a single-edged razor blade [26]. Cells were treated with VEGF (1.2 pmol/l, 50ng/ml) in the presence or absence of NHIgG, aPL (100 μg/ml) or the aPL-mimetic mAb 3F8 (0.3 μg/ml), with or without C44 or 1N11 added (100 μg/ml against NHIgG/aPL, 60 μg/ml against 3F8) in DMEM + 1% LPDS. Twenty-four hours later the cells were fixed with 3% paraformaldehyde (Sigma-Aldrich), permeabilized in 0.2% Triton X-100 (Bio-Rad Laboratories), stained with hematoxylin (Fisher Scientific), and viewed under an inverted microscope (Nikon Eclipse TS100). The number of cells migrated past a 2000 μm length of the wound edge was quantified in a minimum of three 40x magnification fields per well.

### Carotid Artery Reendothelialization

Carotid artery reendothelialization was evaluated following perivascular electric injury in 12–16 week-old male C57BL/6 mice as previously described [26]. Mice were anesthetized by intraperitoneal (IP) administration of avertin, and the left common carotid artery was exposed by an anterior neck incision. Electric current of 4 watts was applied through 2 mm forceps (2 watts/mm) for 3 sec in microbipolar mode (Force 2 Electrosurgical Generator, Valleylab). Mice were recovered and analgesia was provided by IP administration of Bupronex immediately after surgery and 24h later. Seventy-two hours following injury, animals were IV injected with 5% Evans blue dye (Sigma-Aldrich), carotid arteries were harvested, and the area of denudation (which incorporates the dye) was quantified in a blinded manner by image analysis using Scion Image (free software from NIH). To evaluate their impact on reendothelialization, NHIgG or aPL (0.1 mg/mouse), along with C44 or 1N11 (0.1 mg/mouse) were IP injected 24h prior to injury, on the day of injury, and 24h post-injury. These experiments and all others performed in mice were approved by the Institutional Animal Care and Utilization Committee at the University of Texas Southwestern Medical Center.

### Trophoblast Cell Migration and Proliferation

HTR-8/SVneo cells were previously derived from extravillous trophoblasts in the first trimester of human pregnancy and immortalized using simian virus 40 large T antigen [33,34]. These cells exhibit a high proliferation index and they share numerous phenotypic similarities with the parental primary cells [33,34]. HTR-8/SVneo cells were kindly provided by Dr Charles H. Graham (Queen’s University, Canada). The cells were maintained in RPMI 1640 containing 5% fetal bovine serum. Trophoblast migration was evaluated in scratch assays. Cells were grown to near-confluence in 6-well plates, a defined region of cells was removed with a single-edged razor blade, and the cells were incubated for 16h with NHIgG, aPL (100 μg/ml), C44 or 1N11 (200 μg/ml) in the presence or absence of 5% serum added to RPMI1640 containing 1% lipoprotein deficient serum (LPDS) [26]. The cells were fixed with 3% paraformaldehyde (Sigma-Aldrich), permeabilized in 0.2% Triton X-100 (Bio-Rad Laboratories), stained with hematoxylin (Fisher Scientific), and viewed under an inverted microscope (Nikon Eclipse TS100). The number of cells migrated past the wound edge was quantified in a minimum of three 10x magnification fields per well. Trophoblast proliferation was assessed in two ways. First, 24h following equal seeding in 6-well plates at a density of 5 x 10^5^ cells per well, cells were incubated with or without NHIgG or aPL (100 μg/ml) in the absence versus presence of C44 or 1N11 (200 μg/ml) in RPMI1640 + 10% FCS for 24h, and the number of viable cells per well was counted using trypan blue exclusion. The second approach involved BrdU incorporation. Cells were plated at a density of 2 x 10^4^ cells per well in 96-well plates, 4 h later the cells were incubated with or without NHIgG or aPL (100 μg/ml) in the absence versus presence of C44 or 1N11 (200 μg/ml) for 24h, and proliferation was assessed using a BrdU Cell Proliferation ELISA Kit (Abcam) according to the manfacturer's instructions.

### Fetal Resorption

Studies of aPL-induced of fetal resorption were performed in virgin Balb/c mice at 8–10 weeks of age as previously reported [27,28,35,36]. Pregnant females received NHIgG or aPL (10 mg/mouse, IP) on days 8 and 12 of pregnancy, and either C44 or 1N11 (0.5 mg/mouse, IP) on days 8–14. On day 15 fetal resorption rates were evaluated (fetal resorption rate = number of resorption sites/number of surviving fetuses + number of resorption sites).

### *In Vivo* Thrombus Formation

Thrombus formation was assessed using intravital microscopy as described previously [24]. Briefly, 24h following IP injection of NHIgG or aPL (100 μg/mouse), with additional administration of C44 or 1N11 (100 μg/mouse), mice were IV injected with fluorescence-labeled anti—mouse GPIbβ antibody, which targets the platelet GPIb-V-IX complex. Then the mesenteric microcirculation was exteriorized, and thrombosis was induced by ferric chloride (10%, on Whatman filter paper for 45 seconds). Thrombus formation in 80–100 μm arterioles was documented by capturing images (×200 magnification) every 1 second for 40 min with a Regita digital camera (200× magnification; QImaging) attached to a NIKON Eclipse Ti microscope with NIS Element image capturing software. The time required to form a 2000 μm^2^ thrombus and the size of the largest thrombus formed within 6 min were then determined.

### Statistical Analysis

All data are expressed as mean±SEM. Statistical analyses were performed using GraphPad Prizm (Version 6.30). Student’s t test or one-way ANOVA was employed to assess differences between two groups or among more than two groups, respectively, with Tukey's post-hoc testing following ANOVA. In cell culture studies the findings reported were confirmed in at least two independent experiments. P values less than 0.05 were considered significant.

## Results

### 1N11 Blockade of aPL Action on eNOS

**Fig 1:**
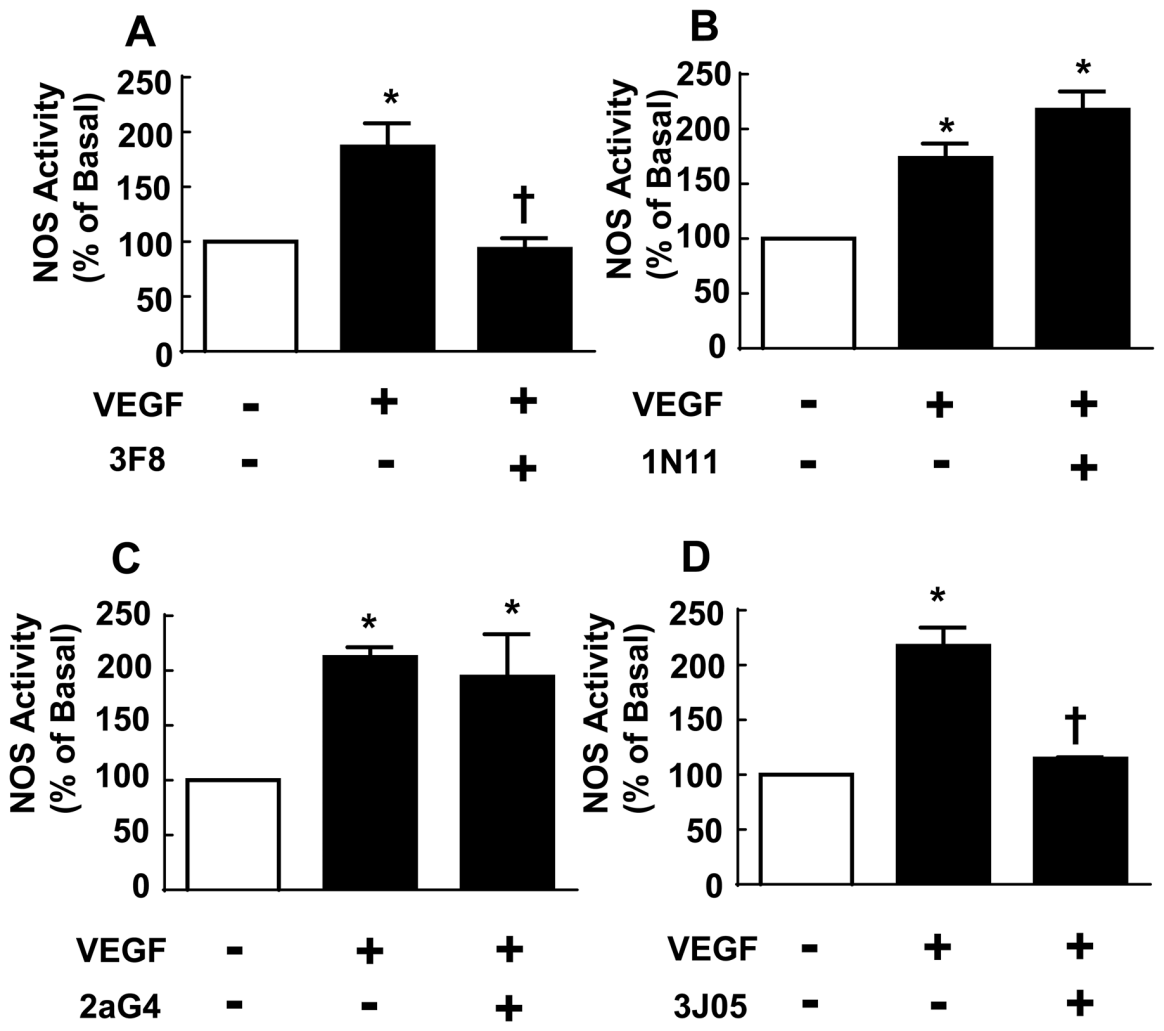
Effect of monoclonal antibodies on eNOS activation by VEGF. Bovine aortic endothelial cells were incubated with vehicle, monoclonal antibody (mAb) 3F8 (A, 2μg/ml), 1N11 (B, 10 μg/ml), 2aG4 (C, 10 μg/ml) or 3J05 (D, 10 μg/ml) for 30 min, and NOS activity was then determined in their continued absence or presence by quantifying [^14^C]-L-arginine to [^14^C]-L-citrulline conversion without (basal) or with added VEGF (100 ng/ml) over 15 min. (N = 3–6, mean±SEM, *p<0.05 vs. no VEGF, †p<0.05 vs. no mAb.).

**Fig 2:**
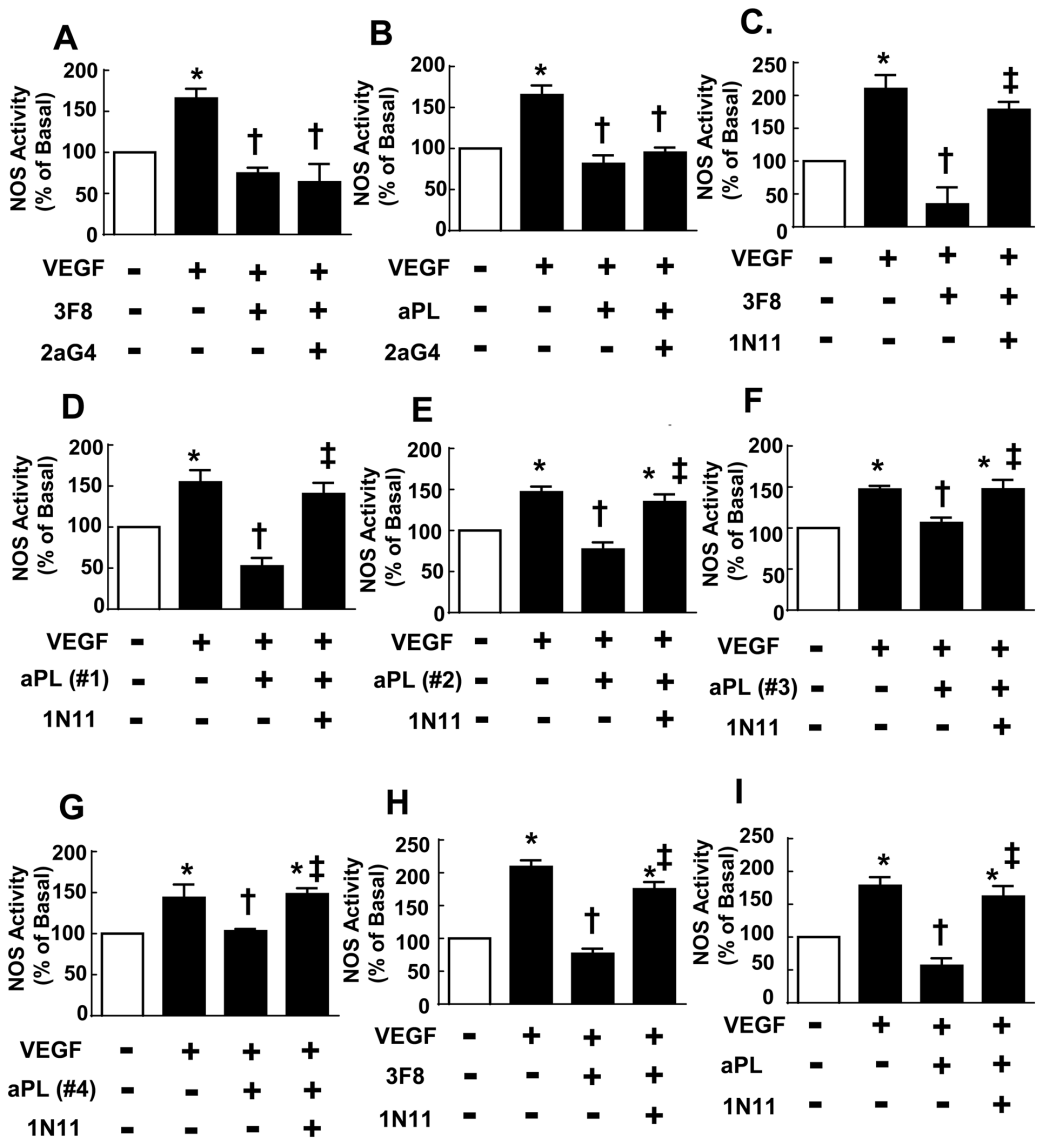
Effect of monoclonal antibodies (mAb) on eNOS inhibition by 3F8 or aPL. The impact of mAb’s 2aG4 (A, B, 200 μg/ml) or 1N11 (C-I, 200 μg/ml) on the antagonism of VEGF stimulation of eNOS by either 3F8 (A, C, H, 2 μg/ml) or aPL (B,D-G, I 100 μg/ml) was determined in bovine aortic endothelial cells (A-G) or human aortic endothelial cells (H, I). This was accomplished by quantifying [^14^C]-L-arginine to [^14^C]-L-citrulline conversion without (basal) or with added VEGF (100 ng/ml) over 15 min. Antibodies were present during 30 min preincubations and during the NOS activity incubations. Studies of 1N11 action on eNOS antagonism by aPL were performed with aPL from 4 different patients. (N = 3–6, mean±SEM, *p<0.05 vs. no VEGF, †p<0.05 vs. VEGF alone, ‡p<0.05 vs. no 1N11.).

Previously we demonstrated that aPL binding to β2-GPI on endothelial cells results in eNOS inhibition, and that this underlies the increase in thrombus formation provoked by aPL in mice [24]. In search of an effective intervention against the actions of aPL that result from the recognition of β2-GPI, we screened a series of mAbs directed against β2-GPI using a highly-sensitive assay of eNOS enzyme activity in intact primary bovine aortic endothelial cells (BAEC). The mAbs tested included 3F8, which mimics the effects of aPL [24], 2aG4 [29,37], 1N11, and 3J05. Evaluating eNOS activation by VEGF, we observed that whereas the mAb 3F8 inhibits eNOS activation (Fig 1A), thereby mirroring the actions of aPL, 1N11 has no effect (Fig 1B). 2aG4 also did not alter eNOS stimulation by VEGF (Fig 1C), whereas the mAb 3J05 caused full attenuation of eNOS activation (Fig 1D). Having no effect on eNOS stimulation themselves, studies of potential 2aG4 or 1N11 prevention of aPL action on eNOS were then undertaken, using either the aPL-mimicking mAb 3F8 or polyclonal aPL isolated from APS patients. 2aG4 did not alter eNOS inhibition by either 3F8 or aPL (Fig 2A and 2B). In contrast, 1N11 prevented the attenuation of eNOS activation by 3F8, as well as eNOS inhibition by aPL isolated from 4 different APS patients (Fig 2C–2G). To determine if the same efficacy is observed for 1N11 in human endothelial cells, studies were done in HAEC. 1N11 caused full reversal of eNOS inhibition by both 3F8 (Fig 2H) and aPL (Fig 2I) in HAEC. Thus, 1N11 does not invoke aPL-like inhibition of eNOS and alternatively it blocks aPL antagonism of eNOS, and this action of 1N11 is apparent in human endothelial cells.

### Molecular Basis for 1N11 Action

**Fig 3:**
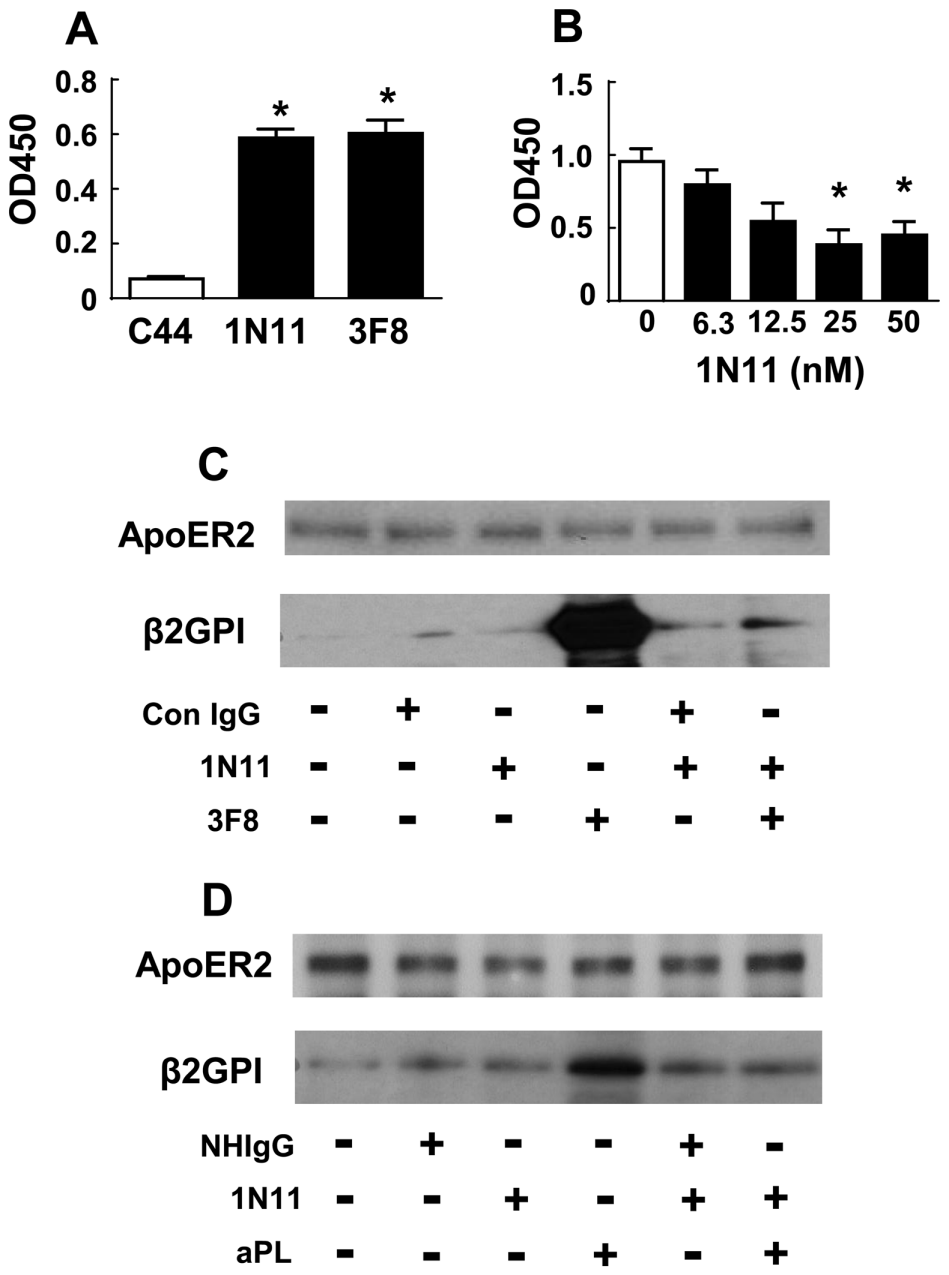
Effect of 1N11 on 3F8 binding to β2-GPI, and on β2-GPI-ApoER2 complex formation induced by 3F8 or aPL. (A) Binding of biotinylated monoclonal antibodies (C44, 1N11 and 3F8) to immobilized human β2-GPI (A) on phosphatidylserine-coated 96-well plates was evaluated (N = 4, mean±SEM, *p<0.05 vs. C44 control.) (B) Increasing concentrations of unlabeled 1N11 were added concurrently with biotinylated 3F8 (10 nM), and 3F8 binding to human β2-GPI was quantified (N = 6–8, *p<0.05 vs. no 1N11). (C) Endothelial cells were incubated in the absence or presence of control IgG or 3F8 (1 μg/ml), without or with 1N11 added (200 μg/ml) for 30 min, ApoER2 was immunoprecipitated, and the presence of ApoER2 and β2-GPI in the immunoprecipitates was evaluated by immunoblotting. (D) Coimmunprecipitation experiments were also performed evaluating β2-GPI and ApoER2 interaction in the absence or presence of NHIgG or aPL (100 μg/ml), without or with 1N11 added. Findings in C and D were confirmed in 2 independent experiments.

To delineate the mechanism by which 1N11 inhibits cellular responses to aPL, we determined whether 1N11 retards the binding of mAb 3F8 to β2-GPI, which triggers an aPL-like response in endothelial cells. Biotinylated 3F8 and 1N11 displayed comparable adherence to phosphatidylserine-immobilized human β2-GPI that was far greater than that of the isotype-matched control IgG C44 (Fig 3A). It was then determined that the binding of biotinylated 3F8 to β2-GPI was displaced by the addition of unlabeled 1N11 in a dose-dependent manner (Fig 3B), with 50% displacement observed at a concentration of 1N11 of approximately 10 nM. Thus, 1N11 blocks the binding of the pathogenic antibody 3F8 to β2-GPI.

The biochemical basis by which 1N11 prevents aPL action was then interrogated. We and others have shown that cellular responses to pathogenic anti-β2-GPI antibody or polyclonal aPL entail the interaction of β2-GPI with ApoER2 [24,38,39]. To first evaluate how 1N11 impacts complex formation between β2-GPI and ApoER2 invoked by the pathogenic anti-β2-GPI mAb 3F8, endothelial cells were incubated with control Ab or 3F8, in the absence or presence of 1N11, ApoER2 was immunoprecipitated in the presence of cross-linker, and immunoblotting was performed for ApoER2 or β-2GPI. Whereas control IgG or 1N11 resulted in negligible coimmunoprecipitation of ApoER2 and β-2GPI, cell treatment with the aPL-mimetic 3F8 yielded marked interaction between β2-GPI and ApoER2, and this was fully prevented by 1N11 (Fig 3C). In like manner, ApoER2 and β2-GPI interaction was prompted by aPL, and this too was attenuated by 1N11 (Fig 3D). Thus, unlike pathologic anti-β2-GPI antibody, 1N11 does not induce β2-GPI interaction with ApoER2, and instead 1N11 inhibits the β2-GPI -ApoER2 complex formation that underlies aPL mechanism of cellular action.

### 1N11 and aPL Attenuation of Endothelial Repair

**Fig 4:**
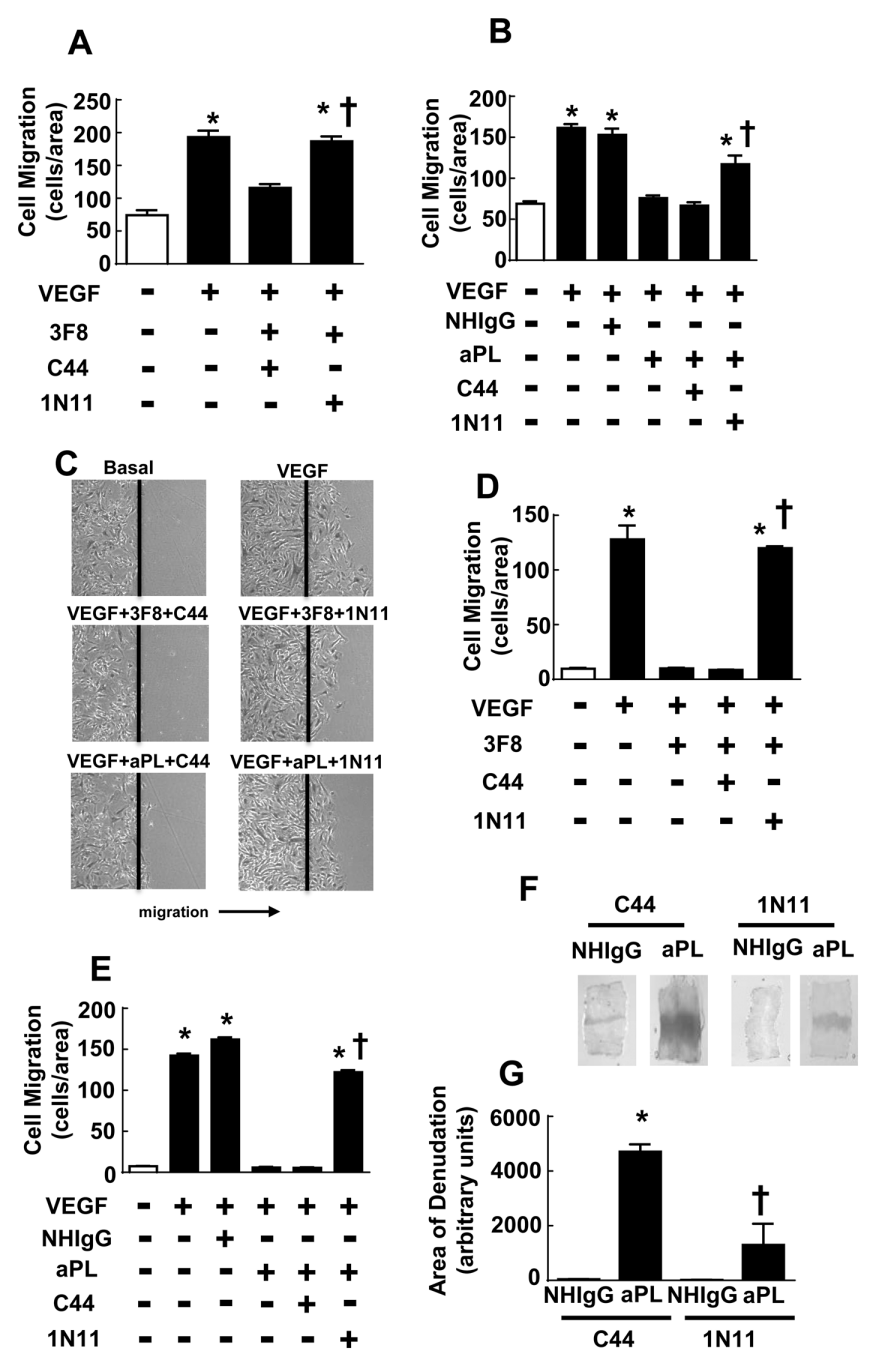
Effect of 1N11 on cultured endothelial cell migration impairment by 3F8 or aPL, and on aPL blunting of carotid artery reendothelialization in vivo. (A) Bovine aortic endothelial cells (BAEC) were incubated without or with 3F8 (2 μg/ml) and either C44 or 1N11 (200 μg/ml), and cell migration induced by VEGF (100 ng/ml) over 16h was assessed by scratch assay. (B) BAEC were incubated with NHIgG or aPL (100 μg/ml) in the presence of C44 or 1N11 (200 μg/ml), and cell migration induced by VEGF (100 ng/ml) over 16h was assessed by scratch assay. (In A and B, N = 8, mean±SEM, *p<0.05 vs. no VEGF, †p<0.05 vs. no 1N11). (C-E) Human aortic endothelial cells (HAEC) were incubated with or without 3F8 (2 μg/ml) or with NHIgG versus aPL (100 μg/ml) in the presence of C44 or 1N11 (200 μg/ml), and cell migration induced by VEGF (100 ng/ml) over 16h was assessed by scratch assay. Representative images of migrating cells are shown in C. (In D and E, N = 5–10, mean±SEM, *p<0.05 vs. no VEGF, †p<0.05 vs. no 1N11). (F, G) Male C57Bl/6 mice (10–12 weeks old) were coinjected with NHIgG or aPL (100 μg/mouse) and either C44 or 1N11 (100 μg/mouse) 24 h before, on the day of carotid artery thermal injury, and 24 and 48 h after injury. Reendothelialization was assessed by evaluating intimal layer Evans blue dye incorporation 72h postinjury. (F) Representative images of the carotid artery intimal surface. (G) Summary data for the area of remaining denudation at 72 h, expressed in arbitrary units. (N = 4–5, *p<0.05 vs. NHIgG, †p<0.05 vs. C44).

Endothelial cell migration plays a critical role in vascular repair and in the maintenance of integrity of the endothelial cell monolayer. A disruption in monolayer integrity is the principle process that initiates the development of the medial hypertrophy and neointima formation that underlie a variety of vascular disorders [40–42]. We previously showed that aPL inhibit cultured endothelial cell migration and reendothelialization of the mouse carotid artery following thermal injury; in association with the attenuated endothelial repair in vivo, aPL caused exaggerated neointima formation, thereby providing an explanation for the increase in non-thrombotic vascular occlusion observed in APS patients [26]. To determine if 1N11 impacts the reparative capacity of endothelial cells when it is adversely affected by aPL, VEGF-stimulated migration was studied in primary bovine aortic endothelial cells. Whereas the pathogenic, aPL-mimetic mAb 3F8 blunted VEGF-stimulated migration in cells coincubated with the control antibody C44, 1N11 treatment afforded normal migration despite the presence of 3F8 (Fig 4A). In parallel, aPL blocked VEGF-induced endothelial cell migration, and this too was reversed by 1N11 (Fig 4B). To determine if the same efficacy regarding migration is found for 1N11 in human endothelial cells, parallel studies were done in HAEC. 1N11 reversed the diminution in VEGF-induced migration caused by both 3F8 and aPL in HAEC (Fig 4C–4E). Thus, 1N11 reverses the detrimental effects of aPL on migration in both bovine and human endothelial cells.

To determine if 1N11 normalizes endothelial repair in the presence of aPL in vivo, carotid artery reendothelialization was studied in male mice. Mice were injected with either NHIgG or aPL in combination with the control antibody C44 or 1N11, and the carotid artery endothelial cell monolayer was denuded by thermal injury. Seventy-two hours later, the remaining area of denudation was determined by intravascular injection of Evans blue dye and evaluation of its uptake by the carotid artery intimal surface (Fig 4F and 4G). In control mice who received C44, there was markedly greater remaining area of denudation with aPL treatment versus NHIgG treatment. The administration of 1N11 decreased the area of remaining denudation with aPL by 75%. These cumulative results indicate that 1N11 prevents the attenuation of endothelial cell migration by pathogenic mAb or by aPL, and that 1N11 administration in mice exerts similar protective effects on endothelial repair in vivo.

### 1N11 and aPL Effects on Trophoblasts and Fetal Resorption

**Fig 5:**
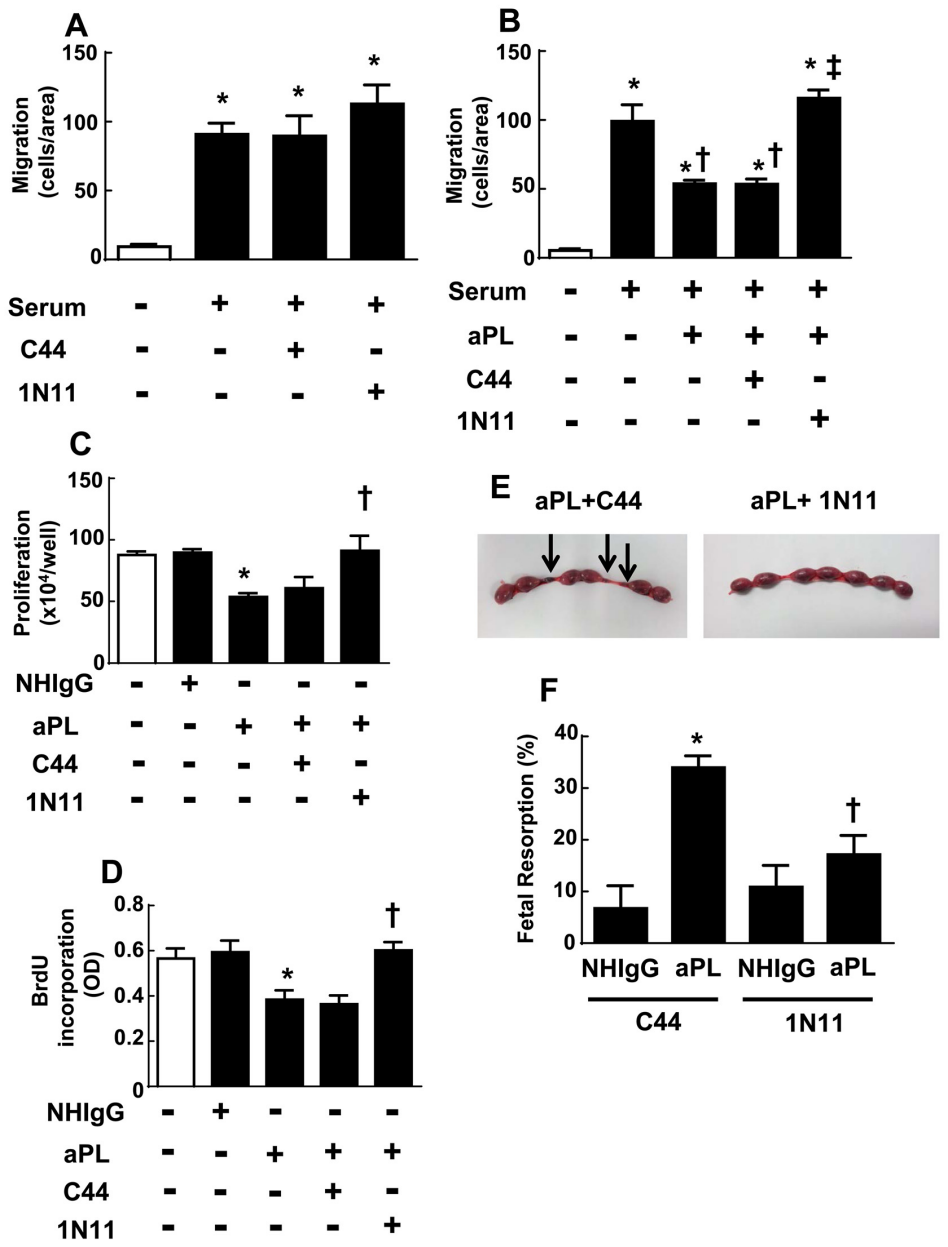
Effect of 1N11 on aPL impairment of trophoblast migration and proliferation, and aPL-induced fetal resorption. (A) The migration of HTR-8 SVneo trophoblasts was evaluated during 16h incubations in the absence or presence of serum (5%), without or with C44 or 1N11 (200 μg/ml) added. (N = 4–8. Mean±SEM, *p<0.05 vs. no serum.) (B) Serum-stimulated migration of HTR-8 SVneo trophoblasts was evaluated without or with added aPL (100 μg/ml), in the absence or presence of C44 or 1N11 (200 μg/ml). (N = 8–16, *p<0.05 vs. no serum. †<0.05 vs. no aPL, ‡p<0.05 vs. C44) (C,D) HTR-8 SVneo trophoblasts were incubated with NHIgG or aPL (100 μg/ml) in the presence of C44 or 1N11 (200 μg/ml) for 24 h, and cell number (C) or BrdU incorporation (D) was quantified. (N = 3–6, *p<0.05 vs. NHIgG, †p<0.05 vs. C44.) (E, F) Pregnant Balb/c mice (8–10 weeks old) were injected IP with aPL or NHIgG (10 mg/mouse) at day 8 and 12 of pregnancy, C44 or 1N11 (0.5 mg/mouse) was administered daily from day 8 to day 14, and fetal resorptions (as indicated by arrows in E) were evaluated at day 15. Fetal resorption rates (number of resorption sites/number of surviving fetuses + number of resorption sites) were calculated (N = 8–9, *p<0.05 vs. NHIgG, †p<0.05 vs. C44.).

Along with vascular disorders, APS patients have greater risk of pregnancy complications including fetal loss [3,11]. Since the obstetric complications are believed to entail aPL actions on trophoblasts, and these are mediated by aPL binding to certain epitopes on β2-GPI [23,43–45], we tested whether 1N11 impacts these processes using the cultured trophoblast cell line HTR-8SVneo [33,34]. First determining that neither the control mAb C44 nor 1N11 inhibits trophoblast migration (Fig 5A), it was then found that aPL attenuation of migration was fully reversed by 1N11 (Fig 5B). Similarly, 1N11 prevented the inhibitory effect of aPL on trophoblast proliferation evaluated by quantifying either cell number or BrdU incorporation (Fig 5C and 5D).

Having demonstrated protective actions of 1N11 on trophoblasts in culture, the effect of 1N11 on fetal loss induced by aPL was evaluated in pregnant mice [27,28,35]. Female Balb/c were injected with NHIgG or aPL on day 8 and 12 of pregnancy, and the control Ab C44 or 1N11 intervention was administered daily from day 8 through day 14. The rate of fetal resorption was then evaluated on day 15 of pregnancy (Fig 5E and 5F). In mice administered the control intervention C44, there was a more than 6-fold increase in fetal resorptions with aPL versus NHIgG treatment. In contrast, in 1N11-treated mice, aPL did not increase the rate of fetal loss above that observed with NHIgG. Thus, 1N11 prevents the adverse effects of aPL on trophoblast cell migration and proliferation, and it suppresses the increase in fetal resorption induced by aPL in vivo in mice.

### 1N11 and aPL Promotion of Thrombosis

**Fig 6:**
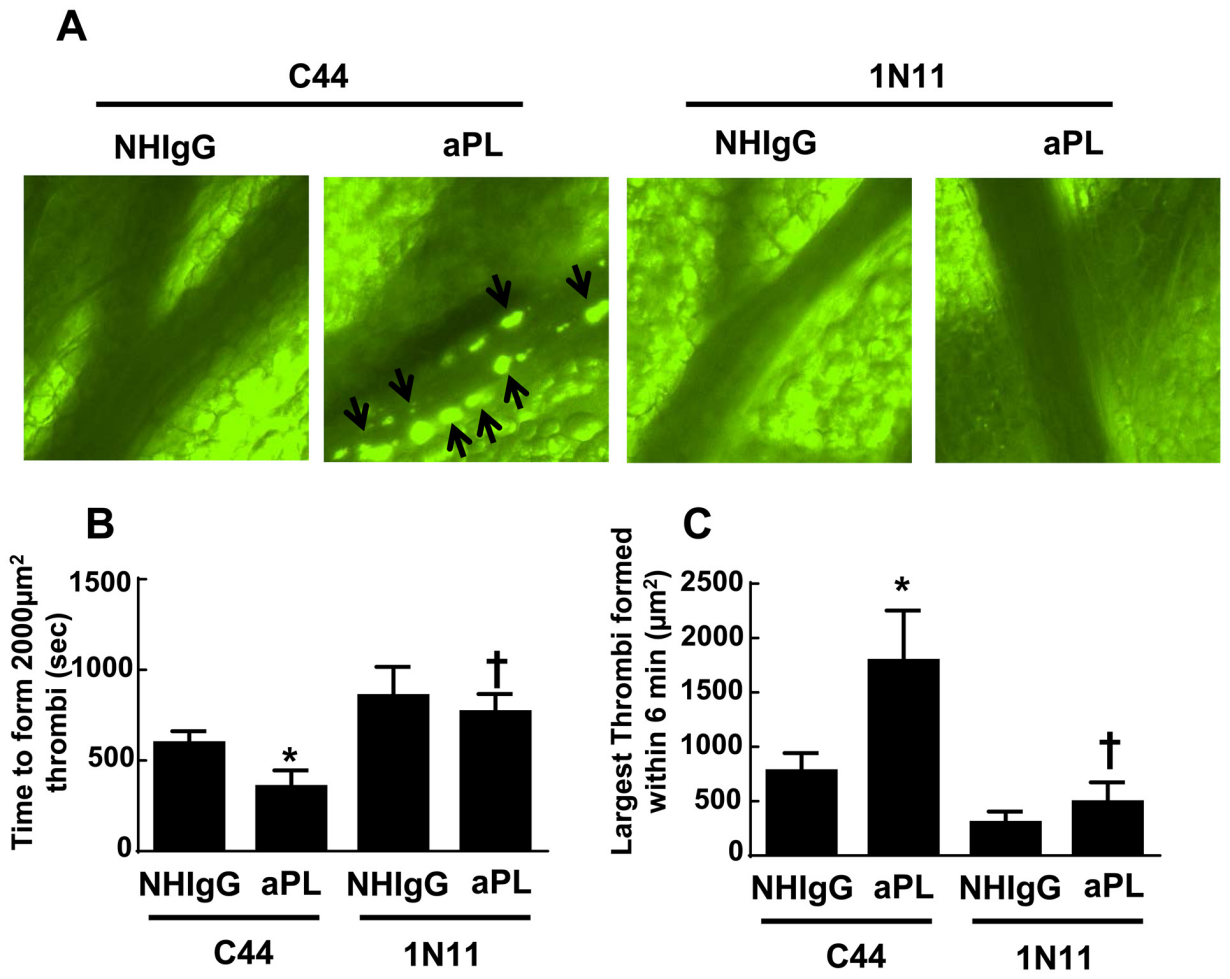
Effect of 1N11 on thrombus formation induced by aPL in mice. (A) Male C57BL/6 mice (5–6 weeks old) received NHIgG or aPL (100 μg/mouse) and either C44 or 1N11 (100 μg/mouse) by intraperitoneal injection. Twenty-four hours later thrombus formation (indicated by arrows) in mesenteric arterioles was evaluated by intravital microcopy. (B) Time required to form 2000 μm^2^ or larger thrombi. (C) Size of largest thrombi formed within 6 min. (N = 6–9, Mean±SEM, *p<0.05 vs. NHIgG, †p<0.05 vs. C44.) The movies corresponding to the images shown in (A) are available at https://figshare.com/s/7f9e28e40bff379c1035.

Remote from pregnancy APS patients suffer from a thrombotic diathesis, and we and others have demonstrated that the exaggeration in thrombosis invoked by aPL and the underlying mechanisms are mediated by β2-GPI and ApoER2 [24,39,46]. We therefore evaluated the effect of 1N11 on aPL-induced thrombosis in mice. Male mice were injected with the control intervention C44 or with 1N11, in combination with either NHIgG or aPL. Twenty-four hours later thrombosis was initiated by the local application of ferric chloride to mesenteric arterioles, and thrombus formation was evaluated by intravital microscopy (Fig 6A–6C). In mice injected with the control intervention C44, aPL shortened the time required to form thrombi larger than 2000μm^2^ in half (Fig 6B). In the setting of C44 control treatment, aPL also increased the size of the largest thrombi formed within 6 minutes by 2.5-fold (Fig 6C). In contrast, 1N11 fully prevented the effects of aPL on both the rapidity and extent of thrombus formation. Thus, 1N11 effectively attenuates the enhancement in thrombosis caused by aPL in mice.

## Discussion

In humans with APS and in experimental models of the disease, aPL cause exaggerated thrombus formation and adverse pregnancy outcomes [1,4], and the involvement of antibodies to certain epitopes on β2-GPI has been demonstrated in numerous paradigms [16,18,47]. Our previous work revealed that polyclonal aPL from APS patients as well as an aPL-mimetic mAb, 3F8, inhibit eNOS, resulting in increased leukocyte-endothelial cell adhesion and thrombus formation in mice [24]. Leveraging a highly-sensitive assay for eNOS activity in intact cultured endothelial cells, we identified an mAb directed against β2-GPI that lacks aPL-related actions but blocks cellular responses to pathogenic mAb or polyclonal aPL isolated from APS patients. The potential in vivo utility of 1N11 was then first demonstrated in studies of aPL antagonism of endothelial repair in mice, which importantly also revealed for the first time that 1N11 alone does not replicate aPL action in vivo. Ultimately it was demonstrated that aPL promotion of thrombosis in mice is fully impeded by 1N11, showing that a clinically important endpoint is dramatically improved by the mAb in an animal model of APS-related disease.

Besides the vascular complications of APS, patients with the disorder can suffer from multiple potential complications during pregnancy including preeclampsia, intrauterine growth restriction, preterm birth, and fetal demise [1–4]. These were originally believed to arise from thrombotic events at the maternal-fetal interface, but more detailed analyses revealed that thrombi are rarely found in the placentas of APS patients [48]. Instead, APS is characterized by attenuated placentation related to reduced trophoblast invasion [48,49], and it has been demonstrated that aPL blunt trophoblast migration and proliferation, which are critical for normal placental development [44,45,50,51]. Complementing our findings regarding 1N11 and the endothelium as a key target cell of aPL action, we determined that 1N11 reverses the detrimental effects of aPL on cultured trophoblast growth and migration. This finding prompted in vivo studies of 1N11 and the pregnancy complications of APS, which can be modeled in mice by either active sensitization prior to pregnancy [52] or passive antibody transfer early in pregnancy or during mid-pregnancy [21,27,28,35,36,53]. Having previously implicated apoER2 in the mechanisms underlying mid- and late-gestation complications of APS [36], we employed a mid-pregnancy passive transfer model and discovered that 1N11 effectively lowers the frequency of fetal resorption invoked by aPL. As such, the intervention may afford a means to better protect the fetus during pregnancies complicated by APS.

In addition to evaluating its potential utility in the clinical disorders that characterize APS, the mechanistic underpinnings of 1N11 action were pursued. Whereas either polyclonal aPL or the pathogenic mAb 3F8 was observed to induce the interaction between β2-GPI and ApoER2 that is prerequisite for aPL actions on target cells [24,39,54], 1N11 failed to do so. Moreover, 1N11 interfered with pathogenic antibody binding to β2-GPI and thereby retarded β2-GPI-apoER2 complex formation. Along with revealing the mechanism of action of 1N11, these results strengthen the concept that the ability of pathogenic antibodies to initiate interaction between β2-GPI and apoER2 is a prerequisite for their provocation of detrimental cellular responses.

Most prior attempts to develop mechanism-targeting interventions for APS have entailed the use of proteins or peptides that mimic sequences within β2-GPI or apoER2 implicated in their actions in APS. Despite displaying efficacy in animal models, none of the peptide-based interventions have been tested in humans [55]. Compared to protein and peptide-based interventions, monoclonal antibody-based therapy is advantageous because whereas the former are challenging due to issues with instability and pharmacokinetics, mAb’s have more favorable pharmacokinetic profiles and they can provide specific and high affinity binding to their targets [56–58]. Recognizing that complement activation is involved in both APS-related thrombosis and fetal loss [59,60], during the performance of the present work another group created a non-complement-fixing antibody to β2-GPI and determined that it prevented the pathologic effects of aPL in mice [61]. Keeping in mind that the readouts entailed cell culture experiments and mouse models of APS-related disorders, the present work shows that 1N11, a fully human antibody that disrupts aPL recognition of β2-GPI, may afford another opportunity to develop an anti-β2-GPI mAb as a therapy.

The potential clinical impact of 1N11 in patients with APS is substantial. aPL are positive in 10% of all patients with deep vein thrombosis, in 11% of all patients with myocardial infarction, and in 13% of all patients with stroke [62]. In addition, despite oral anticoagulation therapy, individuals with a laboratory diagnosis of APS have a 44% likelihood of suffering a thrombotic event within 10 years [63]. As importantly, although the reported rates of bleeding complications vary widely for APS patients receiving anticoagulation, the incidence can be as high as 10% [64]. The impact of APS on pregnancy is also considerable. It has been estimated to complicate 5–7% of all pregnancies, and despite current anticoagulation therapy, the rate of fetal loss in APS is 18%, IUGR occurs in 13–33% of APS pregnancies, and the rate of prematurity is 16–50%, with an average gestational age of 31 weeks [65–70]. Moreover, when anticoagulation is chosen as a means to combat any of the numerous complications of APS, major dilemmas remain regarding the level of anticoagulation to target and the duration of therapy [71]. Spring-boarding from the present discovery of 1N11 as a highly-effective, mechanism-based treatment for APS in a comprehensive series of mouse models of APS-related disorders, clinical studies of 1N11 now warrant consideration.

## Supporting Information

S1 Table Clinical and laboratory features of the APS patients. (PDF)
