# Supplementary material for: Identification of a Monoclonal Antibody That Attenuates Antiphospholipid Syndrome-Related Pregnancy Complications and Thrombosis
Source: PLoS One. 2016 Jul 27;11(7):e0158757. doi: 10.1371/journal.pone.0158757 (PMC4963039; doi:10.1371/journal.pone.0158757)
Supplement: S1 Table — (PDF) [file pone.0158757.s001.pdf]

S1 Table. Clinical and laboratory features of the patients

Patient	Age	Sex	aCL	Anti-β2GPI	Clinical Features
1	57	M	>80 LAPGA	+	Arterial thrombosis, recurrent pulmonary hemorrhage, catastrophic APS
2	50	F	>80 LAPGA	+	Arterial thrombosis, pregnancy losses, catastrophic APS, myocardial infarction
3	32	M	>80 LAPGA	+	Adrenal hemorrhage, skin necrosis, hemolytic anemia, multiple pulmonary emboli, catastrophic APS
4	43	F	>80 LAPGA	+	Arterial thrombosis, digital infarct, leg ulcer
